# Longitudinal Neurocognitive and Pulmonological Profile of Long COVID-19: Protocol for the COVIMMUNE-Clin Study

**DOI:** 10.2196/30259

**Published:** 2021-11-11

**Authors:** Catherine N Widmann, Michelle Wieberneit, Luzie Bieler, Sarah Bernsen, Robin Gräfenkämper, Frederic Brosseron, Carsten Schmeel, Pawel Tacik, Dirk Skowasch, Alexander Radbruch, Michael T Heneka

**Affiliations:** 1 Section Neuropsychology Department of Neurodegenerative Diseases and Gerontopsychiatry University of Bonn Medical Center Bonn Germany; 2 German Center for Neurodegenerative Diseases Bonn Germany; 3 Department of Neurodegenerative Diseases and Gerontopsychiatry University of Bonn Medical Center Bonn Germany; 4 Department of Psychiatry University of Bonn Medical Center Bonn Germany; 5 Department of Neuroradiology University of Bonn Medical Center Bonn Germany; 6 Department of Cardiology, Pneumology and Angiology Internal Medicine II University of Bonn Medical Center Bonn Germany

**Keywords:** SARS-CoV-2, COVID-19, postacute COVID-19 syndrome, cognition, neuropsychology, lung, magnetic resonance imaging

## Abstract

**Background:**

There is a dearth of information about “brain fog,” characterized by concentration, word-finding, or memory problems, which has been listed in the new World Health Organization provisional classification “U09.9 Post-COVID-19 Condition.” Moreover, the extent to which these symptoms may be associated with neurological, pulmonary, or psychiatric difficulties is unclear.

**Objective:**

This ongoing cohort study aims to carefully assess neurocognitive function in the context of the neurological, psychiatric, and pulmonary sequelae of SARS-CoV-2 infection among patients with asymptomatic/mild and severe cases of COVID-19 after remission, including actively recruited healthy controls.

**Methods:**

A total of 150 participants will be included in this pilot study. The cohort will comprise patients who tested positive for SARS-CoV-2 infection with either an asymptomatic course or a mild course defined as no symptoms except for olfactory and taste dysfunction (n=50), patients who tested positive for SARS-CoV-2 infection with a severe disease course (n=50), and a healthy control group (n=50) with similar age and sex distribution based on frequency matching. A comprehensive neuropsychological assessment will be performed comprising nuanced aspects of complex attention, including language, executive function, verbal and visual learning, and memory. Psychiatric, personality, social and lifestyle factors, sleep, and fatigue will be evaluated. Brain magnetic resonance imaging, neurological and physical assessment, and pulmonological and lung function examinations (including body plethysmography, diffusion capacity, clinical assessments, and questionnaires) will also be performed. Three visits are planned with comprehensive testing at the baseline and 12-month visits, along with brief neurological and neuropsychological examinations at the 6-month assessment. Blood-based biomarkers of neurodegeneration will be quantified at baseline and 12-month follow-up.

**Results:**

At the time of submission, the study had begun recruitment through telephone and in-person screenings. The first patient was enrolled in the study at the beginning of April 2021. Interim data analysis of baseline information is expected to be complete by December 2021 and study completion is expected at the end of December 2022. Preliminary group comparisons indicate worse word list learning, short- and long-delayed verbal recall, and verbal recognition in both patient cohorts compared with those of the healthy control group, adjusted for age and sex. Initial volumetric comparisons show smaller grey matter, frontal, and temporal brain volumes in both patient groups compared with those of healthy controls. These results are quite robust but are neither final nor placed in the needed context intended at study completion.

**Conclusions:**

To the best of our knowledge, this is the first study to include objective and comprehensive longitudinal analyses of neurocognitive sequelae of COVID-19 in an extreme group comparison stratified by disease severity with healthy controls actively recruited during the pandemic. Results from this study will contribute to the nascent literature on the prolonged effects of COVID-19 on neurocognitive performance via our coassessment of neuroradiological, neurological, pulmonary, psychiatric, and lifestyle factors.

**Trial Registration:**

International Clinical Trials Registry Platform DRKS00023806; https://trialsearch.who.int/Trial2.aspx?TrialID=DRKS00023806

**International Registered Report Identifier (IRRID):**

DERR1-10.2196/30259

## Introduction

### Background

Prolonged symptoms among patients after resolution of initial SARS-CoV-2 infection are becoming increasingly salient. In addition to long-term respiratory problems and chronic fatigue, patients may also have trouble with concentration and memory as well as psychiatric or neurological complications [1]. These may also occur after an asymptomatic course of the infection; hence, the effect of disease severity remains unclear. One of the most common self-reported symptoms among patients is “brain fog,” which is also denoted as “mental fog” or “clouding of consciousness” [2]. These terms refer to a reduction in alertness and awareness of the environment, an inability to concentrate, and confusion, and can have many causes. Although the term “brain fog” offers an intuitive shorthand for this experience, it is not an official medical diagnosis with clear definitions. In addition, the reported frequency of this experience varies widely depending on the study. These symptoms may ensue myriad other disorders and dysfunctions, including organ dysfunctions, psychological burdens, and disorders such as sleep disturbance and chronic fatigue. Objective data of cognitive performance after acute SARS-CoV-2 infection is, so far, scarce.

An online patient-led survey of COVID-19 patients (N=3762, 78% women, 1.7% nonbinary) sponsored by University College London yielded self-reported fatigue, postexertional malaise, and cognitive dysfunction more than 6 months after initial COVID-19 infection as the most prevalent symptoms from a diverse range of other outcomes [2]. Specifically, subjectively experienced brain fog/cognitive dysfunction was reported by 55.5% of participants and memory problems were self-reported by 50.5% of participants. However, among the small subset of those who reported long-term cognitive or memory difficulties who also had a brain scan, only 13.1% (52/397) revealed neuroradiological correlates.

Such diverse symptoms are proposed to belong to a syndrome now denoted variously as “Long Covid” [3], “persistent post-COVID syndrome” [4], or “post-acute sequelae of SARS-CoV-2 infection” [5], and affected patients have been described as “COVID-19 long haulers” [6]. Although COVID-19 symptoms can now be provisionally classified using the emergency code “U09.9 Post-COVID-19 Condition” from Chapter 22 of the International Classification of Diseases as of January 1, 2021, there are currently no specifications or consensus definitions other than an assumed postpriori connection to acute infection. Such manifestations in COVID-19 patients warrant careful study in an objective, quantifiable, and nuanced manner in the context of possible confounding or contributing factors.

To our knowledge, objective reports of cognitive outcomes are still quite limited. A North American University of Washington study assessed subjective symptoms using an electronic follow-up questionnaire 3-9 months after the onset of SARS-CoV-2 infection. This sample included 177 recovered COVID-19 patients presenting at a specialized clinic (mean age 48.0, SD 15.2 years; 57% women) and a small number of healthy participants (mean age 50.8, SD 15.8 years; n=21, 52% women) [1]. The number of inpatients (16/177, 9.0%) was quite small compared to outpatients (150/177, 84.7%). In addition, 11 (6.2%) patients with asymptomatic courses during the acute infection phase who never presented at the hospital were included in the cohort. The most commonly reported symptoms post-COVID-19 (assessed via self-report) were fatigue and loss of smell in approximately 14% of patients. Brain fog was present in a mere 2.3% of the sample. Around a third of patients reported worse health-related quality of life and approximately 8% reported difficulty with regard to daily activities, most commonly household chores. Persistent symptoms occurred more frequently in those over 64 years of age (13/30, 43%) compared with patients under 65 years of age (42/147, 29.6%). The age structure of the total cohort included comparatively few older patients (30/177, 16.9% of the total cohort), who are commonly known to have more severe courses of COVID-19 and may be at higher risk of later complications [1].

In contrast to these findings, another North American study performed at the Northwestern Memorial Hospital, Chicago, Illinois, analyzed 50 COVID-19 laboratory-positive and 50 COVID-19 laboratory-negative acute cases [2]. All patients were seen at a specialized neurological COVID-19 outpatient clinic between May 13 and November 11, 2020. Both groups had met the characteristic clinical manifestations of COVID-19 and had neurological complications attributed to a suspected SARS-CoV-2 infection for up to 6 weeks, such as headache, numbness, tingling, and fatigue. No demographic differences were found, with an overall average age of 43.2 (SD 11.3) years, and 70% were women. The laboratory-positive patients were examined on average 4.72 (SD 1.83) months after symptom onset, which was approximately 1 month earlier than the laboratory-negative group at an average of 5.82 (SD 1.56) months. Self-reports were used to assess neurological, cognitive, and quality of life symptoms via a computer-based televisit. Across groups, patients reported equal amounts of fatigue (85%) and brain fog (81%), along with depression or anxiety (47%), which were the most frequent symptoms. Cognitive function was assessed in person in a subset of 36 patients using the National Institutes of HealthToolbox v2.1 instrument. Interestingly, the groups did not differ on any measure of executive function, attention, working memory, or processing speed in this study, which may rather reflect the health status of the comparison group (who may indeed have had undetected SARS-CoV-2 infections despite laboratory testing). When positive SARS-CoV-2 patients were compared to matched US normative data, they performed significantly worse on tests of attention and working memory by more than half a standard deviation.

A Zhejiang University School of Medicine in Hangzhou study of a small group of COVID-19 patients (mean age 47, SD 10.54 years; n=29, 3% women) and controls (mean age 42.48, SD 6.94 years; n=29, 59% women) showed discreet difficulties in three parameters of sustained attention in a self-administered, iPad-based online test battery [3]. The tests are part of the MATRICS Consensus Cognitive Battery validated for the Chinese population [4]. The neuropsychological data appear to have been collected in the early postinfection period (ie, 2-3 weeks after infection). It was not reported how many of the cohort were hospitalized or rather seen in an outpatient setting nor how severe the initial course of COVID-19 had been.

Further, an Italian study of 38 patients (mean age 53.45, SD 12.64 years; 29% women) assessed patients hospitalized in Milan between February and April 2020 for complications of SARS-CoV-2 infection [5]. Neuropsychological testing was performed at 4.43 (SD 1.22) months after discharge using the Brief Repeatable Battery of Neuropsychological Tests used in multiple sclerosis research [6]. Patients were screened beforehand with the Montreal Cognitive Assessment (MoCA; cutoff>18.28 points) to exclude those with dementia or cognitive decline. Slowed cognitive processing speed was identified in over 40% of patients; delayed verbal recall impairment was found in 26%, with an overlap in these deficits in 21% of the cohort. More than 60% of all patients performed below the normal cutoff score on at least one cognitive parameter. The average verbal and spatial memory scores were more than half a standard deviation below the norm mean. The average cognitive speed score for COVID-19 patients was more than one standard deviation below the mean of the Italian norm population [6]. Measures of verbal recall were worse for older patients over 55 years (n=20) compared to younger patients with moderate effect sizes, as calculated by us. Importantly, a subanalysis of 33 (87%) subjects of this cohort was selected based on the presence or absence of acute respiratory distress syndrome (ARDS). Those with ARDS (n=12) during hospitalization were compared to those without ARDS (n=21). Despite these small samples, remarkably worse performance for those with ARDS compared to those without were found (based on our effect size calculations derived from data reported in the article: verbal long-term storage (Cohen *d*=1.05), delayed verbal recall (Cohen *d*=0.97), and a challenging variant of a test of speed-dependent sustained attention and working memory task (Cohen *d*=2.63).

In addition to respiratory difficulties, which may be linked to outcomes such as fatigue or brain fog in a very direct manner, neurological complications of COVID-19 themselves may confer a higher risk of incurring cognitive difficulties in both the short and long term [7,8]. Various types of neurological damage and disease may follow COVID-19 with such diverse manifestations as chemosensory disorders, muscular damage, encephalopathy, delirium, coma, meningitis, encephalitis, cerebrovascular diseases, and peripheral and central neuroimmunological disorders [7,8]. Neurological damage has been hypothesized to belong to four types: (1) neurological consequences of pulmonary disease and associated systemic disease (systemic inflammatory response syndrome, sepsis); (2) direct invasion of the virus into the central nervous system (CNS); (3) those caused by postinfectious, immune-mediated complications, including Guillain-Barré syndrome or acute disseminated encephalomyelitis; and (4) peripheral organ dysfunction or failure [1,3]. Indeed, neurological complications of COVID-19 may derive from an amalgam of these four types [8].

Further, psychological distress and psychiatric disorders may directly relate to worse long-term cognitive performance among COVID-19 patients; however, it is known that the general population also presents higher rates of psychiatric burden since the start of the pandemic. Therefore, there is a need to take public health aspects of the COVID-19 pandemic into account in understanding the specific effects of SARS-CoV-2 infection, which requires active recruitment of control groups since the start of the pandemic.

A recent retrospective analysis gives indications of newly diagnosed neurological and psychiatric disease within the first 180 days after SARS-CoV-2 infection (mean age 46, SD 19.7 years; N=236,379, 55.6% women, 0.04% other), which are directly relevant to the phenomenon of “brain fog” and cognition [9]. This cohort comprised mostly nonhospitalized patients (80.4%; mean age 43.3, SD 19.0 years) and around 20% hospitalized patients (mean age 57, SD 18.7 years). Just under 4% were at the intensive care unit (ICU) (mean age 59.1, SD 17.3 years) and around 3% had encephalopathy (mean age 66.7, SD 17.0 years). In total, 33.62% (95% CI 33.17-34.07) of COVID-19 patients had one neurological or psychiatric symptom, with less than half that number being initial presentations (12.84%, 95% CI 12.36-13.33) after infection. Using a propensity-score matching approach, separate COVID-19 cohorts were compared to cohorts with influenza or other respiratory tract infections (RTIs). In the matched comparison of COVID-19 patients (mean age 39.7, SD 18.4 years; n=105,579, 58.6% women) to a sample of influenza patients (mean age 38.6, SD 19.7 years; n=105,579, 57.6% women), a higher hazard ratio (HR) was found for COVID-19 patients for the incidence of any of 14 neurological or psychiatric outcomes (1.78, 95% CI 1.68-1.89). When compared to the matched RTI cohort (mean age 46.0, SD 20.4 years; n=236,038, 56.3% women), the COVID-19 cohort (mean age 45.9, SD 19.7 years; n=236,038, 55.7% women) displayed a significantly higher HR for any first outcome (1.32, 95% CI 1.27-1.36) [9]. Thus, COVID-19 was associated with a higher risk for neurological and psychiatric outcomes compared to rates found in patients with influenza or other respiratory diseases *prior* to the pandemic.

Turning to the relevance of assessing post-COVID-19 sequelae for the world population, other viruses that have run their course, such as severe acute respiratory syndrome (SARS) and Middle East respiratory syndrome (MERS) are valuable sources of information [10]. Specifically, Ellul et al [10] reported CNS diseases among 0.04% of SARS and 0.20% of MERS case counts. The authors extrapolated from the then-current worldwide minimum COVID-19 case count in July 2020 that 1800-9600 patients worldwide were likely to suffer from CNS symptoms. The case count has increased to almost 198 million (as of August 2, 2021) [11], which yields an estimate of anywhere between 79,000 and close to 400,000 patients worldwide who may currently have verifiable CNS symptoms (Table 1).

**Table 1:** Extrapolations of rates of central nervous system (CNS) complications from COVID-19 according to current case counts using estimates from Ellul et al [10] based on severe acute respiratory syndrome (SARS) and Middle East respiratory syndrome (MERS) for selected regions and countries.

Disease	CNS complication base rate (%) [10]	Extrapolated CNS complications from COVID-19, n					
		World^a^	Europe^b^	Germany^a^	United States^a^	India^a^	Brazil^a^
SARS	0.04	79,335	1511	13,774	14,001	12,678	7975
MERS	0.20	396,673	7557	68,872	70,007	63,392	39,877

^a^Based on the COVID-19 web-based dashboard from Johns Hopkins Coronavirus Resource Center accessed August 2, 2021 [11].

^b^Based on the COVID-19 situation update for the European Union/European Economic Area, as of July 30, 2021 [12].

Although epidemiological models of COVID-19 spread show the difficulty of knowing how it will continue (eg, drops due to vaccinations, potential for herd immunity) or what mutations may portend, it is worthwhile to consider hypothetical future population saturation rates and their consequences [13]. Based on the last reported world population estimates from the United Nations of just over 7.7 billion people [14], and extrapolating the base rates of CNS complication in SARS and MERS from Ellul et al [10], we calculated the dimensions of potential CNS damage for the World, Europe, Germany, the United States, India, and Brazil, which are listed for the purpose of illustration in Table 2 (this listing is neither exhaustive nor necessarily reflective of future outcomes in any given region or country). A saturation rate of 30% of the world population by the time the pandemic subsides could lead to between 93 million to around half a billion CNS symptom sufferers worldwide. A 70% saturation rate by the end of the pandemic could lead to between around 220 million to almost 1.1 billion CNS symptom sufferers worldwide. These estimates do not take into account the symptoms that are harder to objectify but are often reported by patients after COVID-19, such as fatigue, “brain fog,” or concentration or memory problems. These estimates also do not account for neuropsychiatric disorders.

**Table 2:** Extrapolations of central nervous system (CNS) complications from COVID-19 based on hypothetical saturation rates of the world population by the end of the pandemic according to base rate estimates using severe acute respiratory syndrome (SARS) and Middle East respiratory syndrome (MERS) data [10].

Population		Population size (millions), n^a^	CNS complications based on hypothetical saturation rates, n	
			30% saturation	70% saturation
**Global**		7794.80		
	SARS (0.04%)		93,537,600	218,254,400
	MERS (0.20%)		467,688,000	1,091,272,000
**Europe**		747.636		
	SARS (0.04%)		8,971,632	20,933,808
	MERS (0.20%)		44,858,160	104,669,040
**Germany**		83.7839		
	SARS (0.04%)		1,005,406	2,345,949
	MERS (0.20%)		5,027,034	11,729,746
**United States**		331.00		
	SARS (0.04%)		39,720,000	92,680,000
	MERS (0.20%)		198,600,000	463,400,000
**India**		1380.00		
	SARS (0.04%)		16,560,000	38,640,000
	MERS (0.20%)		82,800,000	193,200,000
**Brazil**		1380.00		
	SARS (0.04%)		2,550,720	5,951,680
	MERS (0.20%)		12,753,600	29,758,400

^a^Population mid-2020 estimates from the United Nations [14]*.*

In contrast to these comparatively conservative estimates, Taquet et al [9] found a much higher base rate of neurological sequelae of 2.1% following SARS-CoV-2 infections, which may indicate upward of 4,165,061 cases of neurological sequelae (CNS and peripheral nervous system [PNS]) worldwide, with around 70,000 in Germany and 750,000 in Europe alone (see Table 3). This indicates the much higher rates of outcomes extrapolated to several countries and regions for purposes of illustration (the list is not exhaustive). A more detailed breakdown of CNS versus PNS rates is not possible to extract from the reported data due to multiple comorbidities in the sample population.

**Table 3:** Extrapolations based on the latest case counts using the base rates from Taquet et al [9] of first presentation of neurological and neuropsychiatric outcomes after SARS-CoV-2 infection for select regions/countries.^a^

Neurological and psychiatric outcomes	Base rates (%) [9]	Case counts, n					
		World^b^ (N=198,336,258)	Germany^b^ (N=3,778,277)	Europe^c^ (N=34,435,890)	United States^b^ (N=35,003,546)	India^b^ (N=31,695,958)	Brazil^b^ (N=19,938,358)
Neurological	2.10	4,165,061	79,344	723,154	735,074	665,615	418,706
Any psychiatric disorder (mood, anxiety, psychotic)	8.63	17,116,419	326,065	2,971,817	3,020,806	2,735,361	1,720,680
Substance misuse	1.92	3,808,056	72,543	661,169	672,068	608,562	382,816
Insomnia	2.53	5,017,907	95,590	871,228	885,590	801,908	504,440
Any first outcome	12.84	25,466,376	485,131	4,421,568	4,494,455	4,069,761	2,560,085

^a^This table is for illustrative purposes only and is neither exhaustive nor necessarily reflective of the future outcomes in any given country or region.

^b^From the COVID-19 web-based dashboard of Johns Hopkins Coronavirus Resource Center accessed August 2, 2021 [11].

^c^Based on the COVID-19 situation update for the European Union/European Economic Area as of July 30, 2021 [12].

Turning to possible mechanisms of neurological damage that may lead to cognitive problems, two lines of research should be highlighted. One is direct infiltration into the brain, possibly via angiotensin converting enzyme-2 (ACE2) receptors, and another is more indirectly due to acute systemic inflammation. It is known that SARS-CoV-2 viral cells specifically bind to ACE2 receptors, which are expressed in brain structures such as the olfactory bulb, hypothalamus, and limbic system [15]. However, to date, there is still no certainty about how or to what extent SARS-CoV-2 cells may enter the brain directly. Human autopsy studies show little to no direct infiltration of the brain [16,17], although a strong and widespread systemic inflammatory response is apparent [18].

A post-COVID-19 condition may, in fact, reflect more general phenomena that have been documented in the wake of several severe inflammatory diseases and syndromes. Based on our own work among sepsis patients [19], we contend that the hippocampus may be one of the earliest and most affected structures of the brain during chronic or acute inflammatory states due to its particular vulnerability to neuroinflammatory events. Various animal models of acute systemic inflammation show cognitive impairment (especially learning and memory) as well as CNS dysfunction (especially in the hippocampus) after resolution of the initial inflammatory response [20-23]. This may be due to intimate structural connections between the limbic system, which undergirds both the emotional response and several cognitive abilities, and the hypothalamus, which has a central role in the immune-brain connection [24].

As seen among other inflammatory conditions such as sepsis and after major surgery or respiratory conditions such as pneumonia and ARDS, long-term consequences can negatively affect a person’s life in many aspects [25-29]. Several areas of daily activities, including employment, education, housework, and hobbies, can be difficult or impossible years after the initial inflammatory syndrome has been resolved. The psychiatric burden (eg, anxiety, depression, posttraumatic stress disorder) is also known to increase after hospitalization for severe illnesses, which shows associations with cognitive disorders [30,31].

Accordingly, a systematic and thorough study of cognitive ability in the context of neurological and pulmonological complications, activities of daily living, psychiatric health, fatigue, sleep, and other key psychological factors is needed to understand the nature of COVID-19 sequelae. It is therefore pertinent to first examine cognitive abilities among symptomatic and mildly symptomatic/asymptomatic patients to characterize the nature of impairment and its associations with the severity of acute infection. Second, multiple known potentially contributing factors such as CNS or respiratory damage need to be examined. Third, the increased psychiatric burden of the general population and of COVID-19 patients should be addressed, as well as general changes during the pandemic, which requires recruitment of a prospective healthy control cohort. This is the intention of the pilot study, “Long-term Consequences of COVID-19 for Pulmonary and Neurocognitive Disorders (COVIMMUNE-Clin),” outlined herein.

The current understanding of the long-term cognitive sequelae of COVID-19 is limited. Certainly, it remains unclear whether cognitive trajectories are stable, fluctuate, or generally improve or worsen over the long term. There is an urgent need to clarify the extent to which cognitive changes are due to individual or collective experiences or to biological changes from SARS-CoV-2 infection.

### Objectives

We will compare the neurocognitive function and pulmonary sequelae of SARS-CoV-2 infection among patients with asymptomatic/mild and severe cases of COVID-19 after remission of infection as well as in comparison to those of actively recruited healthy controls.

## Methods

### Research Consortium

This is one of three subprojects within a three-pronged research consortium entitled “COVIMMUNE-Studies on immune system function and disease progression of COVID-19.” An investigator at the University Hospital Bonn leads each subproject. The goals of the consortium are to understand the interplay of genetic, epigenetic, and environmental factors that influence innate and adaptive immune responses to SARS-CoV-2, and their links to the broad clinical spectrum of COVID-19 and the associated long-term lung and CNS pathologies.

### Ethical Considerations

This study is being conducted according to the World Medical Association Declaration of Helsinki; the Regulation (EU) No 536/2014 of the European Parliament and of the Council of April 16, 2014, on clinical trials on medicinal products for human use; as well as local ethical research guidelines and research guidelines of University Hospital Bonn (Regulations for ensuring good scientific practice) and the European General Data Protection Regulation (EU) 2016/679 [32-34]. The study protocol was thoroughly reviewed for German data protection compliance by the local data protection officer prior to submission for ethical approval. The study protocol was reviewed by the local Internal Review Board (Medical Ethics Review Board of the University of Bonn Medical Center, ID 511/20) and final approval was obtained on March 10, 2021.

This study is registered at the German Clinical Trials Registry (primary registry trial identifier: DRKS00023806; registration date: March 16, 2021, cross-referenced at the World Health Organization International Clinical Trials Registry Platform).

This pilot study is being conducted exclusively by trained and qualified medical investigators, psychologists, and study nurses who have current Good Clinical Practices certifications.

All participants are informed both in writing (participant information) and verbally by (medical) study investigators regarding all important aspects of the study, including risks and benefits to the individual participant. Participants have sufficient time to process this information and ask any questions prior to providing consent. Each participant gives written consent before taking part in any study-specific procedure. As part of the informed consent process, participants are made aware of the rationale for the study; scope of the study; benefits and risks of study participation; storage and use of data, including data protection measures within the study; data protection rights; the right to withdraw consent at any time; and the right to access their own study data.

### Study Design

This is a monocentric longitudinal prospective cohort study at the University Hospital of Bonn. Assessments will be conducted at three time points (baseline, 6 months, and 12 months) for all participant groups.

### Study Population

A total of 150 participants between the ages of 25 to 75 years will be included in the study. Inclusion and exclusion criteria for the study are presented in Textbox 1. The first cohort (Cohort I) will comprise patients after a SARS-CoV-2 infection with either an asymptomatic course (n=50) or, at most, those who had symptoms of olfactory or taste dysfunction (anosmia, ageusia) only; all other symptoms lead to exclusion from this arm. The second cohort (Cohort II) will include patients after SARS-CoV-2 infection with a severely affected course (n=50), defined as having been admitted to hospital (any ward type) for at least 24 hours due to SARS-CoV-2 infection at any time during the course of the disease. The third cohort (Cohort III) is a healthy control group (n=50) with a similar age and sex distribution to those of the other cohorts, based on frequency matching. For the healthy control arm, a SARS-CoV-2 rapid antibody test will be administered at screening to exclude recent or active infection. We anticipate that the antibody test will reflect those who have developed SARS-CoV-2 antibodies due to SARS-CoV-2 vaccination and this will not exclude them from participation. Further, to exclude those with verbal episodic memory abnormalities before inclusion in the healthy control group, the Hopkins Verbal Learning Test will be administered as a screening method [35]. The exclusion criterion will be long-delayed verbal recall of less than –1.0 SD below the age-specific reference norm value. Additional inclusion criteria for the healthy control group are denial of memory concerns and no known history or current diagnosis of psychiatric or neurological illness.

**Summary of inclusion and exclusion criteria.**

**General inclusion criteria**

- written informed consent
- aged 25 to 75 years
- able and willing to participate throughout the study
- fluent German language abilities

**Cohort-specific inclusion criteria**

Cohort I: Asymptomatic course of COVID-19 (SARS-CoV-2–positive) or mild course (ie, no symptoms other than anosmia and/or ageusia)

Cohort II: severely affected course of COVID-19 (SARS-CoV-2–positive) (ie, requiring hospital stay)

Cohort III: Healthy controls will only be included in the study if they also meet all of the following criteria:

- must perform >–1.0 SD on the Hopkins Verbal Learning Test
- no substance abuse
- no known history of or current diagnosed psychiatric illness
- negative SARS-CoV-2 rapid antibody test at baseline

**General exclusion criteria**

- inability to give informed consent
- any condition that clearly interferes with participation in the study
- any condition that interferes with the clinical or neuropsychological study procedures
- sensory impairment that prevents or significantly interferes with neuropsychological testing
- contraindication for magnetic resonance imaging
- severe or unstable medical condition
- current major depressive episode
- psychotic disorder, bipolar disorder, substance abuse at present or in the past
- known neurodegenerative disorder (Alzheimer disease, Parkinson disease, frontotemporal dementia, Huntington disease, amyotrophic lateral sclerosis)
- vascular dementia, history of stroke
- history of malignant disease

### Recruitment Strategy

Two patient cohorts are being recruited directly via letter. The first cohort derives from the German COVID-19 Case Cluster Study (Heinsberg Study). This study cohort comprises several patients severely affected by COVID-19 who are likely to be at increased risk of subsequent cognitive decline, as well as a large number of mild or asymptomatic cases. Second, SARS-CoV-2–positive patients that have been treated since February 2020 at the University of Bonn Medical Center will be identified by the patient record system or by our COVID-19 outpatient unit, and will then be contacted by letter. In addition, as needed, we will recruit participants for all groups of the study via advertisement on our website, popular social media platforms, and in newspapers. These diverse recruitment methods will indicate an email address for potential participants to contact directly. Those expressing interest in participation will be contacted by our study team via telephone and will undergo a brief telephone screening with the help of a standardized guideline for identifying potential participants.

All participants will be informed and give consent prior to any study-specific procedure.

### Study Procedures

The three groups will be enrolled in a sequential manner to ensure a similar structure with regard to age and sex. The individual assessments will vary from visit to visit. All assessments will take place on one study day. The following will be assessed or carried out for all participants after providing written informed consent and review of the inclusion and exclusion criteria: demographics, medical/surgical history, medical/disease status, neurological examination, blood chemistry, neuropsychological examination, magnetic resonance imaging (MRI), lung function assessment, and (for the healthy controls only) a SARS-CoV-2 rapid antibody test.

### Neurocognitive Examination

The neurocognitive assessment will include comprehensive, standardized, and validated neuropsychological tests, questionnaires, and scales to assess pandemic-related changes on lifestyle, psychological health, sleep, psychiatric symptom burden, and basic and instrumental activities of daily living. Trained, qualified personnel will conduct the neuropsychological assessments at the Department of Neurodegenerative Diseases and Geriatric Psychiatry.

Part of the neuropsychological assessment will be a specially selected, comprehensive computerized assessment with the Vienna Test System (Wiener Testsystem Version 8.15, Schuhfried, Mödling). This will comprise normed, standardized tests for the following domains: complex attention; verbal learning and memory; visual-spatial learning and memory; semantic verbal abilities; and psychological scales for depression, anxiety, and somatic illness.

The primary endpoint of this study is an episodic memory measure due to the posited vulnerability of the hippocampus to effects of systemic inflammation and loss of integrity of the blood-brain barrier in neurovascular and neurodegenerative diseases [36-38]. Since the immune response to SARS-CoV-2 infection itself could increase the risk of developing a cognitive disorder such as mild cognitive impairment or dementia, we chose to include the types of tests commonly used in the diagnostic workup at memory clinics as well as an extensive battery of computerized attentional, executive function tasks.

In addition, we will ask a series of self-report questions regarding changes in memory and general cognitive ability compared to before onset of the acute phase of COVID-19 using a modified version of the Everyday Cognition-12 questionnaire [39]. For healthy controls, we will ask the same questions with the reference point being before the beginning of the pandemic. This is an effort to compensate for the fact that we cannot exclude those with cognitive difficulties prior to SARS-CoV-2 infection in Cohorts I and II.

Other scales used include instrumental activities of daily living, a COVID-19-specific scale of basic activities of daily living (Post-COVID-19 Functional Scale-German) [40], health-related quality of life, and a short screening scale for posttraumatic stress disorder. Owing to established associations between cognitive decline and neuroticism [41], scales will be implemented to assess personality along the dimension neuroticism-extraversion [42]. Further, important lifestyle factors will be explored, including leisure activities and satisfaction with one’s financial situation, perceived loneliness and social isolation, perceived changes in responsibilities at work and at home, and maintenance of intellectual and daily activities [43-46].

Lastly, loss of smelling ability has been connected to systemic inflammation and neurodegenerative disease as well as SARS-CoV-2 infection [47]. Hence, olfaction will be assessed by the Sniffin’ Sticks Test of Smelling Ability (Screening 12 Test, Burghart Messtechnik GmbH) based on the “Odor-Curves-On-Paper” Method [48]. This method enables safe and hygienic testing conditions and has been validated in earlier studies [49].

### Neurological and Physical Assessment

As part of the initial medical workup, medical doctors will assess the medical history and current medications. Concomitant medication, procedures, and medical diagnoses will be documented at each follow-up. In addition, orienting tests of visual, auditory, and olfactory function will be performed. Body weight and height will be measured at the first visit, and heart rate as well as blood pressure will be measured at the first and last visits. At each visit, a neurological examination will be performed. This examination includes analysis of mental status, cranial nerves, motor system, reflexes, sensory system, coordination, and gait assessment.

### Blood Samples

The amount of blood taken at each individual blood draw (baseline and 12-month follow-up) is approximately 18 milliliters. Samples will be drawn by a certified nurse or a medical doctor who is a member of entrusted study personnel for patients and healthy control subjects. Each blood sample includes serum and ethylenediaminetetraacetic acid (EDTA)-plasma, which will be divided into 200-microliter aliquots immediately after sampling and stored at –80°C until biomarker assessment.

### Biomarker Panel

Analysis of blood biomarkers of neurodegeneration will be performed by SIMOA Quanterix assays using fully automated HD-X platforms. Samples will be run in duplicates with a maximum accepted coefficient of variation of 20%. Our design includes sufficient backup material to repeat measures in case of technical failures, if necessary. The biomarker panel includes neurofilament light chain and neuron-specific enolase (NSE). Samples will be analyzed once after conclusion of the baseline recruitment to address analytes with potentially limited long-term storage stability such as NSE [50]. Different sampling methods that might impact the results of blood-based neurodegeneration markers are still under investigation and recommendations might change during the study [51,52]. Our design includes sufficient amounts of both serum and EDTA-plasma to adapt to new findings on preanalytics at the time of analysis and to choose the optimal sample type for each analyte.

### SARS-CoV-2 Rapid Antibody Test

The Acro 2019-nCoV IgG/IgM Rapid Test (Hangzhou Alltest Biotech Co, Ltd, China) is a reliable and rapid chromatographic immunoassay for the qualitative detection of SARS-CoV-2 immunoglobulin (Ig)G and IgM antibodies in human whole blood, serum, and plasma samples. A certified nurse or a medical doctor who is a member of entrusted study personnel will draw the whole blood sample. The SARS-CoV-2 rapid antibody test will be administered at screening and at the 12-month visit only for the healthy control arm of the study.

### Pulmonological and Lung Function Examinations

The pulmonological and lung function examinations will take place at the Department of Internal Medicine (Pneumology) and include forced vital capacity (FVC), forced expiratory volume in 1 second (FEV1), Tiffeneau index (FEV1/FVC), and diffusion capacity for carbon monoxide by the single-breath method. Clinical pulmonological and lung function assessments will include assessment of dyspnea, cough, fatigue, dizziness, chest pain, anxiety, a lung function test, body plethysmography, a 6-minute walking distance test, quality of life questionnaires, and a shortness of breath questionnaire.

### MRI Assessments

At baseline and at 12 months, subjects will undergo MRI according to a standardized clinical protocol at the Department of Neuroradiology on a clinical 3.0 Tesla magnet (Achieva, Philips Healthcare, Best, the Netherlands). Sequences will comprise 3D magnetization-prepared rapid acquisition with gradient echo, fluid attenuated inversion recovery, diffusion, susceptibility weighted imaging, T2-weighted image, and diffusion tensor imaging. Volumetric analysis will be conducted at the Department of Neuroradiology. The acquired MRI datasets will be visually and semiquantitatively assessed by an experienced radiological examiner. In addition, postprocessing of the MRI datasets will be performed using CE-certified artificial intelligence–based software (mdbrain, Mediaire GmbH, Heidelberg, Germany), which allows for automated quantitative analyses of the brain and determines volumes of different brain areas in milliliters as well as age- and sex-adjusted percentiles of a (manufacturer-dependent) normative collective.

### Data Analysis

#### Statistical Analysis

Demographic background, clinical, and biomarker variables will be analyzed in both patient populations and in healthy controls. Additional analyses will be performed for cognitive and neurological data, lung function, MRI, psychiatric burden, and the activities of daily living and health-related quality of life scales. Quantitative variables will be presented in summary statistics of number of patients, mean (SD), and median (range) by appropriate group and time point. Qualitative variables will be described using the frequency count of the events, and the number and percentage of responding patients. The primary and secondary endpoints will be analyzed in an exploratory manner utilizing mixed models and correlational analysis. Statistical analysis will be performed using IBM SPSS Statistics Version 25, 64-Bit Version (IBM Corp, 2017).

Preliminary data based on the Auditory Word List Test reported here were analyzed via multivariate analysis of variance and pairwise effect sizes were calculated based on Hedges *g*, which allows for effect size calculation with different group sizes.

#### Sample Size Calculation

A power analysis was performed on the basis of the primary endpoint: long-delayed verbal recall from the word list recall task of the Vienna Testing System’s Auditory Verbal Learning Test [53]. This parameter was chosen due to the close association of long-delayed recall and hippocampal integrity [54], as well as the hypothesized vulnerability of hippocampal function to complications following COVID-19. In earlier studies, a medium-size difference was observed between healthy controls and patients with dementia [53]. Given the current state of knowledge of the study population, we also feel confident in assuming a medium-sized effect for our study. An a priori analysis of sample size was performed based on a medium effect size *(f*=0.28), a type 1 error probability of 5%, power of 80%, and 3 groups. The outcome parameters yielded a required total sample size of 126 and an estimated actual power of 80%. This resulted in a group size of 42 per arm of the study, which we increased to 50 per arm to account for potential losses to follow-up. To deal with possible SARS-CoV-2 infection of healthy volunteers during the course of the study, 20 more healthy control participants will be recruited for a total of 70 healthy controls.

## Results

### Schedule

Funding for this subproject was granted to the principal investigator (MTH) at the Department of Neurodegenerative Diseases and Geriatric Psychiatry and the coprincipal investigator (DS) at the Department of Internal Medicine II, Cardiology, Pneumology and Angiology at the University of Bonn Medical Center in Bonn, Germany. This subproject aims at fully characterizing and contextualizing neurocognitive performance after SARS-CoV-2 infection within the cohorts studied.

At the time of submission, the study had begun recruitment with the first enrollment on April 8, 2021. As of July 2, 2021, 50 participants (aged 26-70 years, 60% women) have been enrolled into the study. An interim data analysis of baseline information is expected to be completed in December 2021. Study completion is anticipated at the end of December 2022 and final results are anticipated to be published after the first quarter of 2023.

### Verbal Learning and Memory

Preliminary multivariate data analysis of neurocognitive data collected through July 2, 2021, showed statistically significant differences. Group differences between healthy controls (n=28, 67.9% women; mean age 41.18, SD 11.60 years), patients with asymptomatic/mild COVID-19 (n=9, 44.4% women; mean age 46.22, SD 12.06 years), and patients with a severe COVID-19 course (n=13, 53.8% women; mean age 48.62, SD 12.04 years) were found in several parameters: word list learning, verbal recall short-delayed and verbal recall long-delayed, and verbal recognition. These comparisons are preliminary in nature and the current group sizes are not yet sufficient for such analyses.

Hedges *g* effect sizes were calculated to reveal sample size–corrected differences due to unequal group sizes. Healthy controls outperformed both the asymptomatic/mild and severe patient cohorts in verbal learning and memory parameters with performance in the age-adjusted norms. Large effect sizes were found for healthy controls for wordlist learning, short- and long-delayed recall, and recognition when compared with those of the asymptomatic/mild group. Similarly, when comparing the healthy controls to the severe patient cohort, moderate effect sizes were found for long-delayed verbal recall and recognition. Interestingly, there was a small effect size for wordlist learning and short-delayed verbal recall. Long-delayed verbal recall and recognition showed moderate effect sizes between the asymptomatic/mild and severe patient cohorts, with the former performing worse. These effects are expected to become more robust once the target sample size has been enrolled.

### Neuroimaging

Preliminary interim results of baseline neuroradiological MRI examinations of a total of 54 study participants, evaluated by an experienced neuroradiologist, showed no semiquantitative differences in presence, number, and location of medullary lesions and intraparenchymal microbleeds among the different study groups, consisting of healthy control subjects and asymptomatic/mild and severe patients. By contrast, automated measurements of brain tissue volumes or age- and sex-adjusted percentiles tentatively suggest statistically significant group differences in decreased frontal and temporal grey matter brain volumes of patients with severe COVID-19 compared with those of healthy subjects and asymptomatic patients. In addition, patients with severe COVID-19 had statistically significant decreases in measured volume mesiotemporally on both sides compared with that of patients who had an asymptomatic disease course.

## Discussion

### Preliminary Findings

#### Cognitive Impairment

Since this is an ongoing study, only preliminary findings could be reported, which included (tentatively) worse performance of COVID-19 patients compared to actively recruited healthy controls on measures of episodic verbal memory (long-delayed verbal recall and verbal recognition) as well as decreased brain volumes in specific brain areas of patients with severe COVID-19 compared with those of healthy subjects and asymptomatic patients.

In line with these findings, short-term lower cognitive performance based on cognitive screening tests such as the MoCA or Mini Mental Status Examination have been reported in the short term (up to 1 month) after acute infection among those with severe COVID-19 courses, ICU stay, and ARDS (n=12) [55], as well as among patients at a COVID-19 rehabilitation unit (n=87) [56]. Further, another larger study (n=135) utilizing the MoCA screening found cognitive impairment, defined as scores below 26 out of 30 possible points, in 23% of their cohort (29%, 30%, and 3% in patients with severe, moderate, and mild COVID‐19, respectively) [57] at a rehabilitation clinic after discharge. In contrast, a point prevalence study at 4 weeks after acute COVID-19, which used the modified Telephone Instrument for Cognitive Status, found no change in cognition in a cohort of 71 patients [58].

In the moderate term (3-5 months) following acute COVID-19, a few small studies indicated some cognitive impairments; however, almost none of these studies used objective episodic memory tasks for assessment. For example, in a small cohort of COVID-19–recovered patients (n=29) studied 3 months after acute infection, cognitive changes were found in a comprehensive test battery, which included memory tasks; however, only a subset of cognitive parameters were reported and none of these included the memory tasks. Compared to actively recruited healthy controls, a few parameters of continuous attention were meaningfully lowered for patients following acute COVID-19 [3].

Our findings are supported by a few small, medium-term studies, which have also found some cognitive impairments after SARS-CoV-2 infection. At 4 months after infection, self-reported cognitive impairment was found in 20.7% (86/416) of a large cohort, including approximately half intensive and half nonintensive hospitalized patients, interviewed by telephone [59]. In a subset of the same study, 17.5% (73/416) reported subjective memory difficulties [59]. This study included a mixture of objective and questionnaire-based assessments of cognitive difficulties, warranting further, careful, objective study. Our study is also in line with a comparable, yet much smaller cohort (n=29) based on an objective cognitive screening (Screen for Cognitive Impairment in Psychiatry Danish Version, SCIP-D), in which at least some cognitive impairment was detected in 19 (65%) patients [60].

Based on a comprehensive neuropsychological test battery (Brief Repeatable Battery of Neuropsychological Tests), further evidence for cognitive impairment was found at 5 months after discharge in a small (n*=*38) nonintensive sample of patients hospitalized for complications of SARS-CoV-2 infection [5]. Slower processing speed was reported for 42.1% (16/38) of patients, worse delayed verbal recall was found for 26.3% (10/38) of patients, and worse delayed visuospatial recall was reported for 18.4% (7/38) of patients [5].

In contrast, a very large epidemiological study that included individuals who recovered from COVID-19 and concurrently obtained controls utilized a remote-based intelligence assessment, including short-term verbal memory and verbal as well as spatial working memory, but neither long-term verbal recall nor verbal recognition was assessed [61]. They reported no significant group difference in spatial working memory, although this was at the threshold level and was a main effect for visual attention. The timeframe of remote testing was around 3-4 months after hospital discharge. Importantly, this study hinted at a “dose” effect of SARS-CoV-2 infection on cognitive ability based on a stratification of symptoms and type of care needed (from best to worst cognitive performance: symptomatic patients with versus those without respiratory symptoms, those with respiratory symptoms and no home assistance versus those with medical home assistance, those hospitalized with no ventilation versus those with a ventilator) [61].

Taken altogether, the extant evidence is quite mixed and requires systematic and objective examination over the long term (ie, 12 months and more). This study will be the first such attempt.

#### Neuroradiological Findings

Previous MRI studies were mainly based on retrospective hospital data and focused on acute clinical imaging of COVID-19, describing the increased occurrence of (postinfectious) encephalitis [62], acute demyelinating/necrotic hemorrhagic encephalomyelitis–like signal changes [63,64], cerebrovascular disease [65], and Guillain-Barre syndrome [66]. The lack of difference among study groups according to the presence, number, and location of medullary lesions or intraparenchymal microbleeds may be a result of the small number of those included in the preliminary analysis, as recruitment is ongoing. According to the rates of, for example, intracranial hemorrhage, ischemic stroke, or encephalitis up to 6 months after SARS-CoV-2 infection based on Taquet et al [9], we anticipate very few of such manifest cases in this study.

The identification of abnormalities in different brain regions could help clinicians understand the potential neurological sequelae and psychological effects of COVID-19. Quantitative neuroimaging in this study indicated that patients with asymptomatic and severe-type COVID-19 without clinically prescribed specific neurological manifestations or obvious lesions on conventional MRI may still show changes in brain microstructure. Compared with healthy controls and the asymptomatic-type COVID-19, global brain microstructural changes were detected in both the gray and white matter in the severe disease course group. A decrease in cortical thickness and changes in white matter microstructure were more profound and extensive in the severe than in the asymptomatic group, particularly in the frontal and temporal systems bilaterally, but without clear side predisposition in our preliminary cohort. The observed decreased frontal and temporal grey matter brain volumes of patients with severe COVID-19 compared with those of healthy subjects and asymptomatic patients is consistent with other reports of grey matter brain volume loss, even among COVID-19 remitted patients and those with asymptomatic courses [67].

Thus, brain integrity appears to be potentially susceptible to either COVID-19–induced neurotoxicity (ie, direct viral encephalitis) or systemic inflammation induced by the immune response [62], although the exact etiology of the observed changes is still uncertain at this point. However, only few MRI studies with volumetric brain analysis of patients who recovered from COVID-19 are available to date. Moreover, these studies provide inconsistent data, showing that there has been either a decrease in cortical thickness and subcortical volume, particularly in the left frontal and limbic system [68], or an increase in cortical thickness of the olfactory cortices and temporomesial regions, particularly the hippocampal region [69], following COVID-19. Further research is therefore urgently needed to improve understanding of the distribution pattern of potentially COVID-19–induced brain microstructural damage.

### Strengths and Weaknesses

This study is being performed to address the dearth of information regarding long-term cognitive performance among patients who recovered from COVID-19. To date, no studies have employed such comprehensive neurocognitive testing in conjunction with lung function, neurological function, and neuroradiological examinations, and with actively recruited healthy control subjects. Hence, this study is a first step at filling several gaps in our knowledge on the severity of COVID-19 courses and these factors. The first key question to be answered is whether there is any evidence of cognitive impairment over the long term. Another question is whether cognitive performance appears to depend on intact lung capacity or pulmonological health, since these serve the oxygenation of the brain. Our experience with patients so far indicates few lung function difficulties over the long term for a majority of patients. A further question is whether and what changes in brain integrity and volume may undergird reductions in cognitive performance. In addition, several questions regarding olfactory ability in the long term will be addressed by this study: the association of subjective versus objective assessment of smelling ability, associations with cognitive performance, and with emotional well-being.

Next, we assess a series of lifestyle, psychiatric, and psychological health factors that directly or indirectly negatively affect cognitive health. A great strength of this study is that we compare patients to actively recruited healthy control participants who also underwent pandemic conditions so as to ecologically control for potential general negative effects of the pandemic on all of our assessments.

There are several strengths to this study design, which have been thoroughly addressed in the Introduction. To the best of our knowledge, this is the first study to collect cognitive, pulmonary, lung function, neuroimaging, and further psychiatric, personality, and lifestyle data as part of a multidisciplinary, long prospective cohort study. Despite the current large number of publications, only some have focused on cognitive outcomes. Most of these did so only superficially, and none of these reported long-term outcomes (12 months or more). Specifically, the detailed cognitive assessment in this study will deliver comprehensive and objective data that are currently scarce. The results of this exploratory pilot study will deliver important information about the clinical presentation of cognitive symptoms following acute COVID-19 in a broad context and compare them to actively recruited healthy controls who also endured long-term pandemic conditions, which may also affect cognition. Despite being a pilot study, there will be adequate statistical power with a sample size of 150 participants.

There are also several design weaknesses to this pilot study. One is the difficulty in representing all affected age groups. It is known that age groups are affected differently by COVID-19 with respect to the infection rate, mortality, and likely also the extent of prolonged symptoms. Although the age range of those enrolled into the study so far is 26-70 years, participants are on average of middle age for all cohorts. This may reflect the magnitude of symptoms or of concerns among middle-aged patients. We are trying to keep the same age and sex distribution across groups as closely as we can during enrollment. In addition, age will be taken into account in several different ways for data analysis, including transforming the raw scores to age- and sex-normed standard values.

A further potential problem is the heterogeneity in the severe arm of the study. We include those who were in hospital overnight and released the next day as well as those who had longer hospital stays or even intensive care stays. We are aware of the scores of confounding factors this represents, such as organ failure or dysfunction, ventilation, and extracorporeal oxygenation, among others. The intake interview addresses any diagnoses such as organ failure. We will describe the type of hospital stay for the severe arms and will create separate calculations of parameters with and without intensive care patients, since these represent a special group. It is beyond the scope of this study to take individual or specialized treatments at the ICU into account.

The question of what role chronic fatigue syndrome (CFS) plays in cognitive difficulties is central to our study. Diagnoses of CFS at any time prior to or during the study will be taken into account. In addition, several items in our study directly address symptoms of chronic fatigue and sleep disorder, which will be used in comparison of the cognitive and pulmonary data.

It is also unclear whether our findings represent true change among the COVID-19 cohort postinfection given that it is impossible to have a pre-COVID-19 baseline. Likewise, it is not possible to exclude COVID-19 patients with memory problems. We do, however, ask a series of self-report questions regarding changes in memory and general cognitive ability since the acute phase of COVID-19, which includes questions regarding everyday attention, memory, and executive function. For healthy controls, we ask the same questions with relation to the start of the pandemic. This will enable a comparison of subjective perception across groups.

Lastly, participants may become infected (or reinfected) with SARS-CoV-2 during the course of the study. We are not able to conduct polymerase chain reaction testing to assess the current COVID-19 status at the assessment points in this study due to a lack of financing and personnel. We are dealing with this in two ways: one is the blood drop–based antibody testing of the healthy group at baseline and at the 12-month visit to exclude infection within the last few weeks or months (IgG and IgM antibodies), although this is not a perfect method. Second, we ask all participants about current and past (re)infection status at each study visit. Since COVID-19 testing is ubiquitous, we rely on our participants answering this truthfully and to the best of their knowledge.

### Future Research

Future research of cognitive performance after SARS-CoV-2 infection should include stratifications based on age, infection severity, duration since initial infection, organ dysfunction (eg, lung, heart), and perhaps according to required treatments. Age is known to be a key risk factor for cognitive impairment in other syndromes and disease states (such as dementias). Yet, younger patients who acquire cognitive impairment after SARS-CoV-2 infection do so during their most productive years of life. The immediate and mid-term cognitive performance troughs after intensive care are also well-known phenomena [70]. Hence, stratifying according to acute SARS-CoV-2 infection severity (ie, number and type of symptoms, requirement of ventilation, organ dysfunctions, in-home care versus hospitalization versus intensive care) would help to clarify those most at risk of developing cognitive problems. Further, stratifications based on the SARS-CoV-2 strain and number of SARS-CoV-2 *reinfections* in light of mutations such as the B.1.617.2 (Delta) variant [71], which may infect even fully vaccinated patients but may be less lethal, would be important for understanding the impact of SARS-CoV-2 infection on cognition.

In addition, cognitive studies require carefully selected, objective measures based on specialized knowledge of functional cognitive modules and cognitive science to identify the specific neuropsychological functions that are affected. Lastly, myriad factors known to be associated with cognitive ability need to be systematically assessed in addition to cognition to identify their independent contributions and possible interactions. These suggestions for future research will be important for identifying at-risk groups, indications for neuropsychological testing services after SARS-CoV-2 infection, rehabilitation or therapy to those with manifest cognitive impairments, and possibly for targeting neuroprotective therapies during the acute stage of SARS-CoV-2 infection.

### Conclusions

After having thoroughly reviewed the existing literature, to the best of our knowledge, this is the first study to include objective and comprehensive longitudinal analyses of neurocognitive sequelae of COVID-19 in an extreme group comparison of asymptomatic/mild versus severe SARS-CoV-2 infection and actively recruited healthy controls within a broad context of other, pertinent variables. This study will contextualize neurocognitive performance via coassessment of neurological, pulmonary, and a series of psychiatric and lifestyle factors.

The preliminary results of on average poorer verbal learning and verbal memory, along with reduced grey matter and frontal and temporal brain volumes briefly reported herein are quite robust. These findings may change as they are by no means final. Our cognitive and neuroradiological findings also require careful analysis together with other assessments of pulmonary and lung function, neurological, and psychological and lifestyle factors at study completion.

## Abbreviations

ACE2: angiotensin-converting enzyme 2
ARDS: acute respiratory distress syndrome
CFS: chronic fatigue syndrome
CNS: central nervous system
EDTA: ethylenediaminetetraacetic acid
FEV1: forced expiratory volume in 1 second
FVC: forced vital capacity
HR: hazard ratio
ICU: intensive care unit
Ig: immunoglobulin
MERS: Middle East respiratory syndrome
MoCA: Montreal Cognitive Assessment
MRI: magnetic resonance imaging
NSE: neuron-specific enolase
PNS: peripheral nervous system
RTI: respiratory tract infection
SARS: severe acute respiratory syndrome
